# Bullying, Suicide, Substance Use, and Mass Harm: Patterns of Overlapping Threats Reported to a Statewide Anonymous Technology-Facilitated Reporting System

**DOI:** 10.1007/s11121-026-01928-w

**Published:** 2026-05-16

**Authors:** Elyse J. Thulin, Sarah Goodrum, Elizabeth Mather, Justin Heinze

**Affiliations:** 1 Institute of Firearm Injury Prevention, University of Michigan, 1109 Geddes Ave, Ann Arbor, MI 48109 USA; 2 Center for the Study and Prevention of Violence, University of Colorado, Boulder, CO USA; 3 Health Behavior Health Equity, University of Michigan, Ann Arbor, MI USA

**Keywords:** Tip line, Technology-facilitated reporting system, Anonymous reporting, Mental health, Firearms, Adolescent prevention

## Abstract

Technology-facilitated anonymous/confidential reporting systems (TFRS; tip lines) are used in K-12 schools statewide in 34/50 states to enhance youth safety and prevent interpersonal and self-directed harm. Leveraging discreet and digitally relevant reporting modes including Apps, websites and call-in submission options, states receive thousands of tips annually representing a variety of concerns (e.g., suicidal ideation, substance use, weapons access, bullying, potential school shootings). Though overseers of TFRS systems anecdotally note that multiple concerns are often present in a single tip, the unstructured nature and volume of the tip data have been barriers to systems understanding the prevalence and patterns of overlap, and implications of multiple concerning behaviors on severity of the threat. Using mixed methods, this research team manually coded a random sample of just over 10% of tips submitted between November 2019 and May 2023 for concerning behaviors (*n* = 2739; coder inter-rater reliability = 0.92) from a statewide system, then utilized descriptive, bivariate and multivariable regression models and latent class analyses to provide estimates on prevalence, patterns of overlap, and differential effects of multiple concerns reported in a tip with the tip being identified as life-threatening. One-third of tips (32%) described multiple concerning behaviors. The most common concern reported in tips were suicide (21.2%), bullying (20.2%), and aggressive behaviors (19.3%). Regardless of the risk, multiple risk factors in a tip consistently increased the odds that a tip would be classified as life-threatening and require emergency responders. Latent patterns of overlap were best represented by a 6-class solution; patterns of overlap included aggressive behaviors or bullying with identity-based violence; suicide with non-school concerns and substance use; and mass-harm events with more generalized weapon concerns (not related to mass harm events) and other aggressive behaviors. This statewide tip line is used for a variety of reasons, and a third of tips contain reference to multiple concerning behaviors that exacerbated features of risk. The patterns identified in the present analysis can be used to further tailor the implementation of these systems, including informing training materials for students to maximize the effectiveness of these tools.

## Introduction

Contemporaryadolescents are reporting a myriad of health and safety-related challenges; based on national 2023 statistics, 39.7% report feeling sadness or hopelessness in the prior year, 20.4% considered attempting suicide, 16.3% reported being bullied via electronic interactions, 19.2% of youth reported being bullied on school property, and 22.1% reported current alcohol use (About 2025). Additionally, risks features often are concurrent thereby increasing the overall magnitude of potential harm; for example, substance use and suicidality are bidirectionally influential (Alathari 2019). Identification of risk signs and upstream exposures is critical for preventing harmful events, particularly for interpersonal violence, mass harm events, and suicide (Baffsky et al. 2023; Bagge et al. 2013). While screenings in healthcare settings provide one opportunity for identification, community-based systems that enable identification are also critical (Borges et al. 2017). Schools provide important services beyond education; teachers and school administrators often serve as trusted adults (Bryant 2024), universal prevention programs offered through schools can enhance access to services to a wide swath of youth (CDC. 2024; Campbell et al. 2020), and school community members, ranging from students, teachers, staff (such as school counselors, health practitioners, and school resource officers), and family members, can help identify youth who are exposed to or engaging in risky behaviors (Youth 2013). One way that schools are leveraging community member knowledge of potentially risky exposures or behaviors is through technology-facilitated reporting systems (TFRS).

Technology-facilitated anonymous or confidential reporting systems (TFRS), or tip lines, were first created in the U.S. after the 1999 attack at Columbine High School (Cowie 2023). The Columbine Review Commission found that a code of silence existed among adolescents, which discouraged them from relaying their safety-related concerns to school or law enforcement officials (Degli Esposti et al. 2025). Bystander intervention (also referred to as upstanding) (Erickson. 2001), whereby observations of concerning behavior and willingness to report it to school or law enforcement officials, can provide novel and crucial information to prevent youth harm, including mass shooting events (Espelage et al. 2022; Furr 2013). However, engaging in bystander behavior can be challenging (Galán et al. 2021), due to the increased risk of physical harm or negative effects on social relationships (Gavine et al. 2016). Systems that enable delegation bystander behavior, whereby disclose is to a figure of authority who then is responsible for intervention, can alleviate some of those concerns, particularly if the bystander can disclose discretely (Goldstick et al. 2022).

As of 2024, 34 states provide an anonymous or confidential TFRS (Gorzkowski Hamilton et al. 2023). Somewhat akin to interoperability between 988 and 911 that allows for greater collaboration between responding systems (Hale and Viner 2016), statewide TFRS enhance collaboration between multidisciplinary teams involving school members (e.g., school mental health providers, school resource officers), law enforcement, and emergency responding systems to identify and provide relevant responses across a continuum of risky behaviors for youth. TFRS are recognized as a best practice for supporting youth safety at school and beyond with recent evaluations indicating efficacy of these systems for promoting youth wellbeing and improving school outcomes (Holliday et al. 2024; Horowitz et al. 2024; Hsieh et al. 2022). Annual reports from these systems indicate that they receive tens of thousands of tips each year (Langman 2019; Messman et al. 2024), and have prevented mass shootings, interpersonal violence, and youth suicide (Mo et al. 2018). Though originally designed to avert acts of school violence, youth submit information using TFRS across a wide range of concerning behaviors for peers (as bystanders) and themselves (primary help-seeking), ranging from suicide and mental health concerns to bullying and substance use (Gorzkowski Hamilton et al. 2023; Muthén 2018). While most tips are, submitted during times when school is in session, youth utilize these systems outside of school hours particularly for life-threatening situations such as suicide (Nagata et al. 2021). When initiating a tip submission, most TFRS require youth to indicate a reason for their tip (often referred to as the event type) and then youth are asked to explain what is going on and provide additional details in an unstructured field (i.e., open-text field, referred to as the tip narrative). System operators have anecdotally noted multiple safety-related concern are often present in a single tip, although youth are only permitted to select one event type when initiating the tip. While annual reports give insight as to the number of tips and singular event type selected by the youth (Langman 2019; Messman et al. 2024; Nguyen et al. 2023), those numbers are likely an undercount of concerning behaviors tips elicit information on and thus understate their potential impact. However, as thousands of tips are, submitted each year and narratives are complex, unstructured data, the prevalence, patterns, and implications of multiple concerns on necessary response systems, is challenging for these systems to quantify.

Understanding the constellation of risky behaviors reported and how overlap informs severity of tips is important for several reasons. First, identifying the proportion of tips with multiple concerns would enhance existing knowledge on the prevalence and concerns youth submit tips about; currently, all statistics reflect a single event type for each tip, which likely yields an undercount of the total number of concerns that are disclosed through these systems. Second, the patterns of risks reported in tips can reveal information about the groupings of concerning behaviors in the population, which can inform local or regional prevention efforts and strategies. For example, when there are overlap in the concerns, more prevention programs could address these intersecting and inter-related safety issues (e.g., mental health, suicidal ideation, weapons access). Third, recording observed patterns would be important for further tailoring of training and educational programming. Providing examples of the types of overlapping risks to youth and responders could enhance training materials, the quality of tips, and preparation of responders. Finally, multi-concern reports may indicate more serious and life-threatening situations, which has additional implications for responding efforts. A more nuanced and comprehensive understanding of the prevalence and nature of tip lines and the co-occurrence of tip types would yield critical information that could help schools and communities to plan and allocate for the proper resources, staff, and partners available to engage with these threats/incidents and prevent suboptimal responses that could exacerbate harm.

## Current Study

In the current study, we draw tips from the statewide system used in North Carolina (*Say Something*Anonymous Reporting System) to provide novel statistics on the prevalence of single versus multiple concerns reported in TFRS tips, the patterns of overlap of concerns, and associations between multiple concerns and engagement of responding systems. As prior literature has found certain behaviors are likely to be correlated such as bullying and mental health concerns (Ok2Say 2023); bullying, isolation, and mass harm (Bagge et al. 2013); and substance use and suicidality (Alathari 2019); we anticipate similar overlaps in tips. Further, given extant findings that co-occurring risk exposures exacerbate the overall magnitude of harm (Planty 2018), we hypothesize that tips with multiple risk types will be more likely to be classified as higher severity.

## Methods

### Statewide Anonymous Reporting System in North Carolina Schools

In North Carolina, the *Say Something Anonymous Reporting System*was launched in fall 2019 through a collaboration between the North Carolina Department of Public Instruction and the Sandy Hook Promise Foundation. The North Carolina Department of Public Instruction oversees the implementation of state public school laws to 115 local K-12 public school districts representing over 2500 public schools and 200 charter, lab and regional schools (Planty 2020). Implementation of SS-ARS in each school involves annual training of students to identify concerning behavior and reporting that behavior to a trusted adult or through the TFRS, and training of school personnel on how to use the software and leverage the information, submitted to enhance student safety. Once, submitted, tips are reviewed by trained crisis counselors at the American Suicidology Association accredited Sandy Hook Crisis Center. Crisis counselors review the, submitted information and gather additional details from the tipster through an encrypted 2-way chat feature. The crisis counselor determines if the tip requires immediate response (e.g., if it is life-threatening) or if it can be addressed by school personnel during normal business hours and disseminates tips to the appropriate responders. The receiving agency/school district investigates the tip, triages the response, and determines next steps. Finally, once the tip has been addressed, the outcomes of the tip (e.g., school-based discipline, counseling, safety planning, notification of parents, ongoing monitoring) are logged and the tip is classified as being “closed.”

By December 2023, SS-ARS had been implemented in over 95% of the state’s school districts and charter schools, with more than 700,000 students in grades 6 through 12 trained in its use. Between November 2019 and May 2023, over 18,000 tips across a broad set of concerns (e.g., bullying, suicide, possible school shooting threat, substance use) were, submitted. Raw data on the tips indicates that the tips often address mental health concerns (e.g., depression, anxiety, self-harm, suicidality), interpersonal threats (e.g., possible school shootings, bullying), and other risky behaviors engaged in by youth (e.g., substance use, vandalism) or school-related adults (e.g., inappropriate behavior).

### Tip Data

Tip-level data from 2019 to 2023 were extracted from the tip system, and youth identifiers were scrubbed from data prior to any analysis. Tip-level data includes structured and unstructured fields; structured fields include if the tip was identified as Crisis Counselors as being life-threatening and thus requiring emergency responders and the singular event type selected by the person submitting the tip (e.g., the tipster) when initiating the tip, while unstructured data includes a narrative of the problem written by the tipster. From the 18,024 unique tips, submitted between November 2019 and May 2023, 2739 tips were randomly selected and manually coded by research assistants. RAs were trained on the codebook and achieved high inter-rater reliability (0.92). Using a content analysis approach (Rapa et al. 2024), the set of concerning risk types was developed based on the set of pre-existing categories that youth selected from when submitting the tip and modified through iterative review of tips, yielding a total of 15 risks—however, 2 of these were specific to concerning behaviors of adults, and thus not included in current analyses. To enhance clarity, hand-coded behaviors in tips are hereon referred to as risk types, while the tipster indicated concern is the event type.

### Measures

#### Youth-Indicated Event Type

When initiating a tip, youth must select a singular “Event Type” from a predetermined list of 46 concerning events prior to providing a written narrative describing the concern. Examples of event types in this set list are “Bullying/Cyber Bullying,” “Assault,” “Suicide/Suicide Ideation,” “Cutting/Self-Harm,” “Depression/Anxiety,” “Planned School Attack,” and “Threat Against School,” among others.

#### Severity of Tip

For each tip, a crisis counselor determined if the tip requires emergency responders such as police or emergency medical services (1) or is sent only to a school team to address during normal school hours (0).

### Hand-Coded Risk Types

#### Aggression

Aggression was defined as once-off aggressive acts, including physical interactions, physical fights, and verbal, written, or aggressive acts intended to inflict harm to one’s emotions, state of mind, or ability to act autonomously (e.g., coercion).

#### Antisocial Behaviors

This includes behaviors such as theft, truancy, vandalism, animal cruelty, and suspected drunk or reckless driving. This does not include substance use or fighting.

#### Bullying

Reflecting standards set by the Centers for Disease Control and Prevention (Reinke et al. 2025) bullying was defined as a behavior which occurs multiple times where there is a power dynamic between the victim and perpetrator. For the present analysis, bullying included verbal, psychological, physical, cyberbullying, and unspecified forms of bullying (e.g., if the tipster said someone was being bullied, but didn’t specify details).

#### Concerning Behaviors Not at School

Concerns outside of school include child abuse, physical abuse between two adults (e.g., elder abuse), and domestic violence.

#### Dating Violence

Dating violence was any form of physical, verbal or cyber violence or aggression between current and/or former dating partners.

#### Gang-Related Concerns

Gang-related concerns included gang-affiliated behaviors or other reference.

#### Identity-Based Violence

Identity-based violence was defined as hate speech, hate crime, derogatory slurs, and discrimination towards a group of people with a shared identity.

#### Mass Harm

Mass harm included concerns about a potential mass shooting, a suspected planned school attack (broadly), or reference to shooting up a school.

#### Mental Health

Mental health included any mention of depression, anxiety, post-traumatic stress disorder, anger, or stress, which may include loss of interest in activities or other normal life events, trouble sleeping, changes to eating, withdrawal/isolation. The mental health code did not include suicide.

#### Sexualized Violence

Sexualized violence encompassed non-consensual physical contact and coercive verbal or psychological behavior with sexual content, excluding long-term abuse.

#### Substance-Related Concerns

This code was defined as tips with reference to any addictive and/or illegal substances, such as tobacco, vaping, alcohol, cannabis, illicit drugs, using prescribed drugs that are not theirs/not as prescribed, and across different actions/modes (e.g., distribution, use, planned parties).

#### Suicide

Suicidality was defined as self-harm behaviors or explicit mention of suicide or suicidality.

#### General Weapon Concern(s)

Generalized weapon concern referred to carriage or use of weapons such as guns and knives, outside of mass-harm reasons.

### Analytic Approach

#### Qualitative Comparison with Youth Identified Event Type

In addition to confirming high inter-rater reliability of manual coding completed by the research assistants (IRR = 0.92), we further evaluated the robustness of our coding by examining overlap patterns between the singular youth selected event-type and the hand-coded risk factor(s) present in each tip. Specifically, we compared the qualitative codes generated by the research team to the singular event type indicated by the youth using descriptive statistics. Research assistants only reviewed the unstructured narrative field and thus were blinded to the structured event-type field selected by the youth. The findings from this step are presented in Qualitative Coded Concerning Behaviors Relative to Youth-Indicated Event Type.

#### Statistical Analyses

Descriptive statistics were used to produce prevalence estimates of counts of risks in tips overall and by risk type. Logistic bivariate and multivariable regression models were used to examine if the count of risks within a tip was associated with severity of the tip overall and stratified by the tip type.

Latent class analysis (LCA) was used to identify unobserved subgroups within tips with two or more concerning behaviors (i.e., a restricted subsample of tips with two or more concerning behaviors). Rare event types (those occurring in fewer than 5% of tips) were not included in the LCA analyses due to model stability issues. Model fit for each solution was evaluated using the Akaike Information Criterion (AIC), Bayesian Information Criterion (BIC), sample-sized adjusted BIC, entropy, Vuong-Lo-Mendel-Rubin Likelihood Ratio Test (VLMR-LRT) as well as class structure interpretability and theoretical relevance (Safe2Say 2022). While each of these fit statistics are common for model evaluation, there is nuance in their differences; specifically, AIC tends to overfit more simplistic models, BIC performs best with more simple models while sample-size adjusted BIC reduces the penalty for small samples and thus is able to assess models with larger number of parameters but fewer observations; in the case of differences, the sample-size adjusted BIC has been found to be the most robust measurement statistic (Safe2Tell 2023). Entropy is an indicator of class separation from 0 to 1, with higher values of entropy indicating less overlap in classes; however, high entropy could be a result of an overfit model, and thus other measures of model fit are important[(Salas-Wright et al. 2016)]. Finally, VLMR-LRT formally tests the difference between iterative class sizes (e.g., between k and k—1, where k is the number of classes), with the null hypothesis being no difference. After selecting the class solution determining the best-fitting model based on these criteria, latent classes were interpreted by examining the item-response probabilities, which describe the likelihood of providing a particular response given membership in a class. Descriptive and comparative analyses were conducted in Stata 19, and latent class analyses were conducted in Mplus 8 (Sen and Bradshaw 2017).

## Results

Roughly 10% of the 2739 hand-coded tips (*n* = 301) were tips that did not reflect relevant concerning behaviors, such as generalized school complains (e.g., tips about school rules during COVID-19, the organization of the car pickup line from school), and were not included in present analyses. Concerns about an adult that were not related to a youth were also removed (*n* = 91). Of the 2438 tips on concerning behaviors related to youth, 1,658 (68.0%) of tips referred to only one concerning behavior, and 780 (32.0%) had information on two or more behaviors. Of those with two or more concerns, the average number of risks reported in tips was 2.3 (median = 2, range = 2–8).

### Prevalence of Concerns Reported in Tips

Across the 2438 tips, the most common reported behaviors in tips included suicide (*n* = 581, 21.2% of tips), bullying (*n* = 553, 20.2%), and aggression (*n* = 528, 19.3%), while the least common behaviors were gang-related concerns (*n* = 16; 0.6%), dating violence (*n* = 23, 0.8%) and anti-social behaviors (*n* = 78, 2.9%; Table 1). Stratified by singular versus multiple risks, for tips with one risk, bullying was the most common concern (*n* = 315, 19.0%), followed by suicide (*n* = 306, 18.5%) and substance use (*n* = 250, 15.1%). In tips with multiple risks, mental health (*n* = 310, 39.7%), aggression (*n* = 281, 36.0%), and suicide (*n* = 275, 35.3%) were most common, and were present in over one third of tips. Correlation between risk types in tips with 2 or more risks were low to moderate, ranging from − 0.379 to 0.455 (Appendix Table 6).

**Table 1:** Distribution of hand-coded risk types in tips

Hand-coded risk type	Prevalence of hand-coded risk type across all 2438 tips *n* (%)	Prevalence of hand-coded risk type in 1,658 tips with 1 concerning behavior *n* (%)	Prevalence of hand-coded risk type in 780 tips with 2 + concerning behaviors *n* (%)	Prevalence of tips with 2 + hand-coded risk types *n*/*N* (%)	Mean (x̄), median (M), and range of number of risks in tips with 2 + hand-coded risk types
Aggression	528 (19.3%)	247 (14.9%)	281 (36.0%)	281/528 (53.2%)	x̄ = 2.5 M = 2, [2,8]
Anti-social behaviors	78 (2.9%)	48 (2.9%)	30 (3.8%)	30/78 (38.5%)	x̄ = 2.2, M = 2, [2,4]
Bullying	553 (20.2%)	315 (19.0%)	238 (30.5%)	238/553 (43.0%)	x̄ = 2.4, M = 2, [2,8]
Concerning behaviors not at school	116 (4.2%)	21 (1.3%)	95 (12.2%)	95/116 (81.9%)	x̄ = 2.8, M = 3, [2,7]
Dating violence	23 (0.8%)	8 (0.5%)	15 (1.9%)	15/23 (65.2%)	x̄ = 3.1, M = 3, [2,8]
Gang-related concerns	16 (0.6%)	3 (0.2%)	13 (1.7%)	13/16 (81.3%)	x̄ = 2.8, M = 3, [2,4]
Identity-based violence	191 (7.0%)	56 (3.4%)	135 (17.3%)	135/191 (70.7%)	x̄ = 2.5, M = 2, [2,8]
Mass harm	221 (8.1%)	137 (8.3%)	85 (10.8%)	85/221 (38.5%)	x̄ = 2.6, M = 2, [2,7]
Mental health	402 (14.7%)	92 (5.6%)	310 (39.7%)	310/402 (77.1%)	x̄ = 2.4, M = 2, [2,8]
Sexualized violence	256 (9.4%)	138 (8.3%)	118 (15.1%)	118/256 (46.1%)	x̄ = 2.6, M = 2, [2,8]
Substance use	350 (12.8%)	250 (15.1%)	100 (12.8%)	100/350 (28.6%)	x̄ = 2.7, M = 2, [2,8]
Suicide	581 (21.2%)	306 (18.5%)	275 (35.3%)	275/581 (47.3%)	x̄ = 2.4, M = 2, [2,8]
Weapon (not mass harm)	155 (5.7%)	37 (2.2%)	118 (15.1%)	118/155 (76.1%)	x̄ = 2.5, M = 2, [2,7]

### Qualitative Coded Concerning Behaviors Relative to Youth-Indicated Event Type

To check for construct validity of the team codes (beyond established inter-rater reliability) and demonstrate the potential for under-reporting based on youth-indicated event types (whereby youth are only able to select a singular event type), we compared the single-event type selected by the youth (as a function of TFRS design) to the hand-coded risk types for the hand-code category of suicidality and the youth event types of “Suicide/Suicide Ideation” and “Cutting/Self-Harm.” Across the 2438 tips, the research team identified suicidality to be present in 581 (23.8%) of tips while youth indicated the singular event type to be “Suicide/Suicide Ideation” for 240 of the tips (9.8%) and “Cutting/Self-Harm” in another 264 tips (10.8%). Within the 581 tips that were coded by the research team to be about suicidality, 41.8% (*n* = 243) of tips were identified to be about “Cutting/Self-Harm” by the youth, 37.2% (*n* = 216) as being about “Suicide/Suicide Ideation,” and 10.0% (*n* = 58) about “Depression/Anxiety” (Appendix Fig. 1). For tips indicated by youth to be about “Suicide/Suicide Ideation” (*n* = 240), 216 (90.0%) of the tips were identified by hand-coders as being related to suicide; 136 of the 240 (56.7%) were identified by the research team as having only one risk, 91 (37.9%) as having two or more risks, and 13 (5.4%) were not identified as having any risk. Of the 13 that the team identified as not having a risk type, further review of those tips found that three tips were seeking advice about dating or friendship challenges that did not align with any of the risk categories, nine tips had insufficient information in the narrative to be interpretable, and one was misclassified by the team and should have been coded as suicidality. Within the 136 where the youth selected “Suicide/Suicide Ideation” as the event type where the research team identified only one risk present, the research team indicated suicide as the risk for 130 (95.6%); 5 of the remaining 6 tips were identified by the research team as mental health related, and 1 tip was identified as aggression. In the tips identified by youth as being about “Suicide/Suicide Ideation” where the research team identified two or more risks reported, 86 (94.5%) were coded by the research team for suicide. Additional risks coded by the research team were other mental health concerns (*n* = 59, 64.8%), a concerning behavior not at school (*n* = 15, 16.5%), access or broader reference to a weapon (*n* = 13, 14.3%), substance use (*n* = 12, 13.2%), aggression (*n* = 12, 13.2%), bullying (*n* = 6, 6.6%), sexualized violence (*n* = 3, 3.3%), a suspected mass harm event (*n* = 2, 2.2%), and identity-based violence (*n* = 1, 1.1%). A similar breakdown of hand-coded risks within tips where youth selected “Cutting/Self-Harm” is present in Appendix Fig. 1.

### Comparison of Severity of Tip by Multi-concern Status Within Type of Concern

Across all risk types (determined by hand-coding), the odds of a tip requiring immediate, life-saving response was significantly higher in tips with two or more risks (OR = 1.51, *p* <.001). When stratified by each risk type, the presence of multiple risks (versus a singular risk) in a tip significantly increased the odds that a tip would be classified as life-threatening and require emergency responders for all risk types except for mass harm, generalized weapon concerns, and suicide; Appendix Table 7). When all risk features were accounted for in a multivariable logistic regression model, the count of risks present (ranging from 1 to 8) remained associated with greater odds of requiring immediate life-saving response (OR = 2.78, *p* <.001; Table 2). In the first multivariable model that contained all risk types, tips classified as having risk factors of mass harm (OR = 4.88) and suicide (OR = 2.3) were significantly at greater odds of being classified as lief-threatening and needing immediate response. In iterative multivariable models, the risk types that are theoretically most likely to require emergency responders (and thus may skew modeling) of mass harm, suicide and generalized weapons were iteratively removed. However, in all models, the number of risks in a tip consistently remained associated with greater odds of the tip being considered life-threatening and requiring emergency response.

**Table 2:** Multivariable logistic regression of count and risk type on life-threatening classification of tip

Variable	Model 1 OR [95% confidence interval, *p*-value	Model 2 OR [95% confidence interval, *p*-value	Model 3 OR [95% confidence interval, *p*-value	Model 4 OR [95% confidence interval, *p*-value
Count of risk types in tip	2.784, [1.807, 4.289], *p* <.001	13.588, [9.055, 20.39], *p* <.001	8.306, [5.937, 11.621], *p* <.001	5.871, [4.479, 7.697], *p* <.001
Aggression	0.254, [0.137, 0.471], *p* <.001	0.052, [0.031, 0.088], *p* <.001	0.093, [0.060, 0.144], *p* <.001	0.106, [0.069, 0.163], *p* <.001
Bullying	0.086, [0.041, 0.180], *p* <.001	0.018, [0.009, 0.034], *p* <.001	0.029, [0.016, 0.054], *p* <.001	0.035, [0.019, 0.064], *p* <.001
Concerning behaviors not at school	0.432, [0.217, 0.859], *p* =.017	0.088, [0.046, 0.172], *p* <.001	0.132, [0.069, 0.251], *p* <.001	0.193, [0.106, 0.351], *p* <.001
Identity-based violence	0.100, [0.032, 0.317], *p* <.001	0.021, [0.007, 0.063], *p* <.001	0.036, [0.012, 0.104], *p* <.001	0.044, [0.015, 0.128], *p* <.001
Mental health	0.546, [0.325, 0.918], *p* =.022	0.112, [0.070, 0.179], *p* <.001	0.158, [0.101, 0.247], *p* <.001	0.232, [0.158, 0.343], *p* <.001
Sexualized violence	0.109, [0.045, 0.268], *p* <.001	0.022, [0.010, 0.052], *p* <.001	0.037, [0.017, 0.083], *p* <.001	0.046, [0.021, 0.100], *p* <.001
Substance use	0.161, [0.082, 0.317], *p* <.001	0.033, [0.018, 0.060], *p* <.001	0.052, [0.030, 0.091], *p* <.001	0.066, [0.038, 0.113], *p* <.001
Weapon (not mass harm)	*	0.205, [0.112, 0.374], *p* <.001	0.397, [0.239, 0.66], *p* <.001	–
Suicide	2.294, [1.358, 3.876], *p* =.002	0.470, [0.331, 0.668], *p* <.001	–	–
Mass harm	4.881, [2.672, 8.917], *p* <.001	–	–	–

^*^Omitted due to collinearity

### Latent Class Modeling

The 780 tips that had two or more tip types were included in latent class analyses. The AIC and sample-size adjusted BIC fit statistics were best for an 8-class solution, BIC was lowest for a 6-class solution, entropy was highest for a 6-class solution, and VLMR-LRT supported classes 2–6 were better fitting than the prior class size (Table 3). As the 6-class solution had highest interpretability and statistical support, it was selected as the final class structure. Classes were defined by the risk types that had the highest probability per class (Table 4). For example, class 1 has a moderate probability of having information about a weapon (0.74), mass harm (0.57), and/or aggression (0.45). In three of the classes, the probability that tips in that class would have information pertaining to a risk type was 1.0. Specifically, information pertaining to substance-use was certain in class 2, information pertaining to bullying was certain in class 4, and information pertaining to identity-based violence was certain in class 6.

**Table 3:** Fit statistics by number of classes

Class solution	AIC	BIC	Sample size-adjusted BIC	Entropy	VLMR *p*-value
1-class	7812.80	7859.39	7827.64	–	–
2	7280.37	7378.21	7311.53	0.81	*p* <.005
3	7052.18	7201.28	7099.66	0.82	*p* <.005
4	6948.50	7148.85	7012.30	0.86	*p* =.011
5	6899.31	7150.91	6979.44	0.89	*p* <.005
6	6808.68	7111.53	6905.12	0.92	*p* <.05
7	6808.95	7163.06	6921.72	0.88	*p* = 0.18
8	6758.11	7163.47	6887.20	0.90	*p* = 0.06
9	6764.93	7221.54	6910.34	0.90	*p* = 0.32

**Table 4:** Probability of risk type by latent class

Event type	Class 1	Class 2	Class 3	Class 4	Class 5	Class 6
Substance use	0.00	1.00	0.09	0.03	0.00	0.03
Bullying	0.10	0.08	0.03	1.00	0.00	0.00
Sexualized violence	0.06	0.19	0.00	0.16	0.59	0.04
Aggression	0.45	0.35	0.04	0.32	0.66	0.95
Non-school problems (home, family)	0.00	0.18	0.26	0.03	0.22	0.00
Mental health	0.11	0.05	0.89	0.29	0.42	0.10
Suicide	0.19	0.27	0.92	0.10	0.21	0.00
Mass harm	0.57	0.07	0.01	0.00	0.00	0.04
Weapon (not mass harm)	0.74	0.14	0.03	0.02	0.00	0.03
Identity-based violence	0.02	0.03	0.02	0.31	0.02	1.00

### Class Structure

The largest class (class 4; *n* = 216, 27.4%) had a high probability of containing information pertaining to bullying, a moderate probability of information about aggression, and a moderate probability of information on identity-based violence (Table 5). The second largest class (class 3; *n* = 208, 26.4%) had a high probability of information about suicide, a high probability of other mental health concerns, and a moderate probability of information about non-school-related problems. In the third largest class (class 1, *n* = 127, 16.1%), tips had a moderate probability of having information about weapons, mass harm, and aggression. The fourth largest class (class 5; *n* = 98, 12.4%) had a moderate probability of information on aggression, sexualized violence, and mental health concerns that were not suicide. The fifth largest class (class 2; *n* = 79, 10.0%) had a high probability of information pertaining to substance use, a moderate probability of aggression, and a moderate probability of suicide. The final and smallest class (class 6; *n* = 61, 7.7%) was defined by having a high probability of information about identify-based violence and aggression.

**Table 5:** Class descriptions and size from 6 class solution

Latent class #	Latent class distribution *n*%	Description
1	127 (16.1%)	Moderate probability of weapon, mass harm, and aggression
2	79 (10.0%)	High probability of substance use, moderate probability of aggression and suicide
3	208 (26.4%)	High probability of suicide and other mental health concerns, moderate probability of non-school-related-problems
4	216 (27.4%)	High probability of bullying, moderate probability of aggression and identity-based violence
5	98 (12.4%)	Moderate problem of aggression, sexualized violence and mental health concerns (not including suicide)
6	61 (7.7%)	High probability of identity-based violence and aggression

## Discussion

Technology-facilitated reporting systems are implemented statewide in 68% of US states (Gorzkowski Hamilton et al. 2023), but little empirical evaluation exists to determine how youth use these systems (Sheftall et al. 2016). While annual reports use structured data fields including the singular event-type selected by the youth from a predetermined set of events when they initiate a tip to provide descriptive statistics, TFRS staff note anecdotally that tips often contained multiple forms of concerning behaviors that are not reflected in these descriptive statistics. Identifying the prevalence of tips with multiple concerning behaviors, the types of behaviors commonly overlapping, and patterns of overlap have important implications for interpreting existing data on TFRS use, implementation considerations including training, and responding systems.

Just under one third of tips (32%) had two or more concerning risks described in the unstructured text written by the person submitting the tip. Regardless of the specific concern(s) listed, the presence of multiple concerns increased the likelihood that the tip would be classified by a trained Sandy Hook Promise Crisis Counselor as life-threatening and requiring emergency response. This aligns with research noting the exacerbating effect of co-occurring risk features on health and behavioral outcomes (Sinha et al. 2021). While overlapping risks consistently exacerbated severity of the tip, it is also important to note that there are inherent differences in severity and urgency of certain risks (e.g., mass harm and suicidality versus bullying). Future work that identifies contextualizing information may be helpful in determining when emergency services are*not* required would be a beneficial future step to determine resource allocation. Additionally, determining when emergency services are not required can reduce the potential for trauma related to police interactions.

The patterns of overlap in concerning behaviors reported in tips aligns with extant literature that demonstrates that risk factors often cluster (Smith 2018), and simultaneously demonstrates the heterogeneity of risk exposures contemporary youth face and importance of contextualizing behaviors reported in these systems. Across multiple TFRS systems, bullying or aggressive interpersonal behaviors are top tip types reported (Langman 2019; Messman et al. 2024), which may correspond to the prevalence of these behaviors, as 19% of high school students report being bullied at school in the prior year (About 2025). In the present study, we found that bullying and aggressive behaviors were also highly common across tips. However, when examining overlap, we found important contextualizing features of these common behaviors that are not always reportable in the annual reports. Notably, in two classes, identity-based violence, including hate speech, derogatory language, or discriminatory behaviors, were present. Demonstrating that TFRS are capturing this type of behavior is important given its high prevalence and early emergence; 20.1% of Black children aged 10 and 11 reported unfair treatment due to their race by other students (Stein-Seroussi et al. 2025), while an even greater percent of teens report identity-based bullying, with youth holding multiple marginalized identities having the highest rate of victimization (Stein-Seroussi et al. 2023). As minoritized youth are more likely to experience victimization and experience higher rates of mental health concerns including suicidality, suicide attempts, and death by suicide, identifying violence perpetrated against these students is critical.

Another common behavior reported through TFRS are concerns related to suicide and self-harm. Suicide is a leading cause of death for contemporary US youth, with 40% of youth reporting persistent feelings of sadness or hopelessness, 20% seriously considering a suicide attempt, and 9% reporting an attempt (About 2025). In the current study, we found overlap in suicide concerns with non-school related problems. Capturing issues beyond the school building is important for enhancing youth safety. The location of youth suicide is most often residential (Stueve et al. 2006), and prior research has shown that youth turn to TFRS for reporting life-threatening information about peer suicide at times when school is not in session, such as in the middle of the night (Thulin et al. 2026; Nagata et al. 2021). Equally important is the finding of overlap between substance use and suicidality in reporting, as other researchers have shown that substance use, and particularly alcohol use, can be a determining factor for an individual attempting suicide (Thulin et al. 2024). However, one of the major limitations of tip lines is insufficient information, submitted in an initial tip to be acted upon (Muthén 2018). While many anonymous TFRS, including the one used in North Carolina, contain features for encrypted 2-way communication between the tipster and the Crisis Counselors, tipsters are not required to engage in further communication and direct follow up with tipster is often impossible given the anonymous nature of the system. Ensuring students are trained to submit comprehensive, relevant information is particularly important for suicide-related concerns so that support can be extended to youth who are struggling.

Another important overlap was for the concerns of weapons, mass harm, and aggression. The first TFRS was created in Colorado in 2004, following the Columbine mass shooting (Cowie 2023). Firearm injury is the leading cause of death of adolescents (Thulin et al. 2025), and over the past 20 years school shootings have increased in frequency(Vaismoradi et al. 2013). Mass shooting events cause substantial direct and indirect trauma and are highly reported on in mass media (Wong et al. 2021). While the substantial effects of a single mass harm event are substantial and preventing these crimes is of high importance, it is also critical to prevent other firearm-related injuries at schools given that 99% of school shootings are acts of interpersonal aggression (and not mass harm events) (Vaismoradi et al. 2013). Our coding scheme distinguished between generalized weapons concerns and suspected mass harm risks, and the overlap of aggression with generalized weapon concerns and suspected mass harm risks indicates that both mass-harm and interpersonal violence concerns are being reported through TFRS.

The findings from the present study represent one state system, but given similarities in the software engineering across platforms and observed data reporting metrics (Gorzkowski Hamilton et al. 2023), are applicable to most statewide TFRS. While an undercount of tip types limits interpretation on what these systems are being used for and how often, functionally, the staff who run TFRS note that it is important for students to select one event type when they submit a tip so that the responder has initial information, which can be particularly important if the student does not write a lot of information in the open-text portion of the tip submission. However, the information gleaned from the current study is still useful for informing how systems structure their data capture and can be useful to tailor student training. Expanding reporting capabilities within TFRS systems to reflect overlapping tips is an important step to ensure data reliability and validity. As each tip is closely reviewed by a trained staff member, modifying the underlying software system to have a place where the staff member indicates additional concerns while closing out the tip would be a positive step. Additionally, on the student side, tailoring training to address which event type should be selected if there are multiple concerns may be important to help students use systems most efficiently and ensure accurate system statistics. This is particularly important given tips reflecting multiple risks are more likely to be life safety than tips with only one concerning behavior.

The pattern of multiple tips and tip overlap have training implications for both TFRS providers, as well as school community members, themselves. To date, there is limited data available on how TFRS staff are trained to respond to tip concerns including how TFRS staff determine the priority level of a tip and successfully elicit additional information (Gorzkowski Hamilton et al. 2023; Holliday et al. 2024). However, prior research has indicated that training on TFRS is critical to their uptake and effectiveness to prevent youth harm (Horowitz et al. 2024). Our results point to the value of collecting additional or related concerns, perhaps with target follow-ups when tipsters raise an issue that may accompany others. Similarly, training for students, staff, and community members on how and when to use systems, likely has implications for how much tipsters share when communicating their concerns. Sharing examples or encouraging the reporting of additional risks may help contextual concerning behaviors, which in turn could lead to better or more tailored responses for the party of concern. Finally, future research examining other tip-characteristics, such as the relationship between the submitter of the tip and the person the tip is about, could yield other important insights that would be relevant for training and implementation.

### Limitations

This study has several important limitations. First, the data are drawn from a single state from the 2019 to 2023 period. While other research has shown similarities in tipping trends across state systems (Thulin et al. (2026), Nagata et al. 2021), other work has noted important differences in TFRS design and implementation that likely influences the types of concerns students submit tips about across systems (Gorzkowski Hamilton et al., 2023). Therefore, the trends by risk type may differ by location. However, the finding that multiple risks are present in a substantial proportion of tips is likely generalizable, particularly given consistent anecdotes noted by TFRS staff across systems. Related to differences in structure of state systems,*Say Something* Anonymous Reporting System does not have mandated reporting by risk type; rather, trained Crisis Counselors ascertain the severity of the tip to determine when it is necessary for emergency services to be engaged. Systems with automated elevation practices may therefore have differences in trends. However, we note the importance of human review and nuanced elevation of risk, for both resource allocation reasons and to prevent unintended harms from the system (e.g., over-response resulting in trauma). Finally, given data privacy and sanitation steps to remove youth identifiers in the present data, we cannot identify different tips on the same situation. While certain risks may be more likely to receive multiple tips for the same unique incident and thus be over-represented, our use of a random sample of tips for hand code likely minimizes this problem.

## Conclusion

Students submit a variety of concerns to this statewide tip line, and one in three tips contains information about multiple concerning behaviors. This finding suggests that TFRS may be important sources for population level understanding of the prevalence of risk factors youth experience. Moreover, overlap in concerning behaviors increases the likelihood that the tip will be considered severe and necessitating emergency responders. Patterns of overlap often represent exacerbating risk factors and important contextualizing information that is useful in addressing the risk. The findings underscore the value of enhancing data capture within systems to accurately portray the types of risks youth use these systems for, but also highlight the potential shortfall of TFRS if designed or their usage is normalized in such a way where only limited or structured information is solicited.

## Figures and Tables

**Fig. 1:**
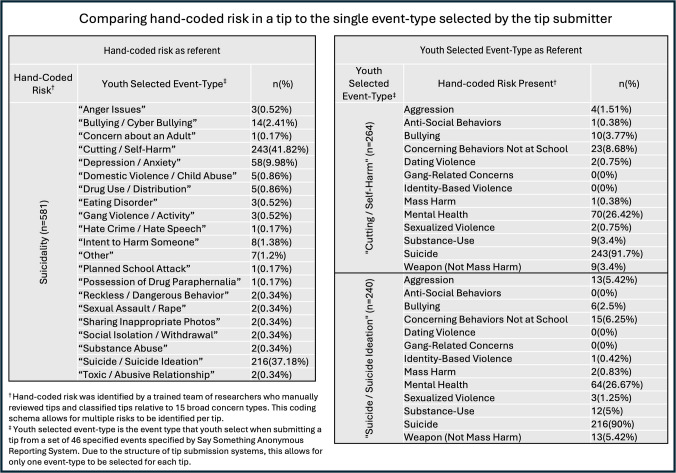
Comparing hand-coded risk(s) in a tip to the single event-type selected by the tip submitter

## Data Availability

Data from this study is available upon request from the primary author.

## Appendix

Figure 1

**Table 6:** Correlation matrix of hand-coded risk types

	Aggression	Bullying	Concerning behaviors Not at School	Identity-based violence	Mass harm	Mental health	Sexualized violence	Substance use	Suicide	Weapon (not mass harm)
Aggression	1.000									
Bullying	− 0.079	1.000								
Concerning behaviors not at school	− 0.148	− 0.180	1.000							
Identity-based violence	0.178	0.184	− 0.137	1.000						
Mass harm	0.045	− 0.164	− 0.109	− 0.119	1.000					
Mental health	− 0.353	− 0.166	0.080	− 0.256	− 0.199	1.000				
Sexualized violence	0.051	− 0.003	-0.009	− 0.104	− 0.080	-0.165	1.000			
Substance use	− 0.037	− 0.143	0.097	− 0.114	− 0.069	-0.154	0.004	1.000		
Suicide	− 0.379	− 0.338	0.136	− 0.318	− 0.178	0.390	− 0.219	0.022	1.000	
Weapon (not mass harm)	0.007	− 0.185	− 0.130	− 0.141	0.455	− 0.268	− 0.092	− 0.051	− 0.139	1.000

**Table 7:** Distribution and bivariate association of count of risks and life-safety status, stratified by risk type

Risk type (count of tips with risk type)	Prevalence of life-safety status in tips by risk type *n* (%)	Bivariate logistic regression of count of risks present in tip on life-safety status* OR, S.E., *p*-value
Aggression (*n* = 528)	42 (8.0%)	2.178 (0.32), *p* < 0.001
Bullying (*n* = 553)	13 (2.4%)	2.626 (0.61), *p* < 0.001
Concerning behaviors not at school (*n* = 116)	23 (19.8%)	1.632 (0.36), *p* = 0.028
Identity-Based Violence (*n* = 191)	4 (2.1%)	16.10 (16.48), *p* = 0.007
Mass Harm (*n* = 221)	117 (52.9%)	0.749 (0.11), *p* = 0.054
Mental Health (*n* = 402)	90 (22.4%)	1.603 (0.22), *p* < 0.001
Sexualized Violence (*n* = 256)	8 (3.1%)	2.776 (0.70), *p* < 0.001
Substance Use (*n* = 350)	18 (5.1%)	2.667 (0.53), *p* < 0.001
Suicide (*n* = 581)	195 (33.6%)	1.048 (0.10), *p* = 0.632
Weapon (not mass harm) (*n* = 155)	56 (36.1%)	0.945 (0.17), *p* = 0.753

*Note: Referent is tips classified as not being life-threatening. For each bivariate logistic model, life-safety status is the independent variable, and tip type is the dependent variable. Tip types were modeled independently
